# Professional nurses’ perceptions regarding clinical competence of community service nurses in North West province, South Africa

**DOI:** 10.4102/hsag.v26i0.1688

**Published:** 2021-10-28

**Authors:** Kholofelo L. Matlhaba, Abel J. Pienaar, Leepile A. Sehularo

**Affiliations:** 1 School of Nursing Science, Faculty of Health Sciences, North-West University, Mahikeng, South Africa; 2 Department of Health Studies, College of Human Sciences, University of South Africa, Pretoria, South Africa; 3 School of Health Sciences, Department of Psychology, University of Venda, Thohoyandou, South Africa; 4 Graduate and Research Department, Shifa College of Nursing, Shifa Tameer-e-Millat University, Islamabad, Pakistan

**Keywords:** clinical competence, community service, community service nurse, perceptions, professional nurse

## Abstract

**Background:**

South African Nursing Council requires nurses who successfully complete their training to perform a year of community service before obtaining registration as professional nurses (Regulation 425). Community service for health professionals was introduced as a strategy to retain newly qualified professionals within the country. The premise is that community service for newly graduated nurses gives them the opportunity to develop skills and acquire knowledge critical in their professional development.

**Aim:**

To explore and describe the perceptions of professional nurses as the supervisors of community service nurses (CSNs) during their 12 months of community service.

**Setting:**

Selected hospitals of the North West province, South Africa.

**Methods:**

A qualitative, exploratory and descriptive design was used. The study was conducted between September and November 2018 at three public hospitals in the North West province. Through purposive sampling, 15 professional nurses who supervise CSNs participated in the study. Data were collected in three focus group discussions using semi-structured questions. All focus group discussions were recorded and transcribed for analysis. Data were analysed using Pienaar’s four steps of thematic analysis.

**Results:**

Three themes emerged: perceptions of clinical competence, challenges impacting clinical competence and suggestions to improve clinical competence.

**Conclusion:**

It is suggested that even though the majority of CSNs were perceived to be competent and capable of working independently, they still required supervision and mentorship to refine their competency. Furthermore, the study reported similar challenges noted from previous studies that were perceived to be affecting CSNs’ ability to deliver quality health care, and therefore recommendations for improvement were made.

**Contribution:**

The study contributed to the developed the clinical competence evaluation tool which will be of benefit to the future community service nurses in the province.

## Introduction

Chapter 3 of the *Constitution of the Republic of South Africa* (RSA) in 1996 states that the government is constituted as national, provincial and local spheres that are distinctive, interdependent and interrelated (RSA 1996). The principle of co-operative government and intergovernmental relations specifies that the government must secure the well-being of the people of the Republic (s 41 (1) (b)). These principles served as a basis for the introduction of community service in South Africa (SA) (RSA 1996), whereby all health professionals are expected to perform community service.

Community service refers to the compulsory service that health professionals are expected to render at public health care facilities after successful completion of training before they can be registered as professional practitioners (Karamchand & Kistnasamy 2017). Frehywot et al. (2010) define community service for health professionals as a law or policy of a particular country that regulates the compulsory deployment and retention of a health professional in an underserved or rural area of that country for a certain period. The first group of health professionals to perform community service in South Africa were the medical doctors in 1998 (Department of Health 2006). The second group were dentists in 2000 followed by pharmacists in 2001. Other health professionals included those from clinical psychology, dietetics, environmental health, occupational health, physiotherapy, radiography and speech, language and hearing therapy followed in 2003 (Department of Health 2006). The *Nursing Bill* was promulgated into the *Nursing Act* (No. 33 of 2005), and the community service for nurses was implemented in 2008 (South African Nursing Council 2007).

The policy regarding community service is explained in section 40 of the *Nursing Act*, 2005 (No. 33 of 2005) and in the *Regulations Relating to Performance of Community Service* published in Government Notice No. 765 of 24 August 2005 with the objective of ensuring the equitable distribution of health workers, especially for rural and underserved populations. In addition to providing a service, the purpose of community service is to provide community service nurses (CSNs) with the opportunity to develop their skills, knowledge, experience and clinical competence. Furthermore, it is expected that such community service also encourages CSNs to remain in the public service, particularly in rural and underserved areas (Hatcher et al. 2014). Therefore, newly qualified professional nurses as South African citizens seeking registration with the South African Nursing Council (SANC) for the first time are required to perform community service for a period of 1 year at a designated public health institution (SANC 2007).

During the 12 months of community service, CSNs work under the direct and indirect supervision of professional nurses. Literature including that of Makua (2016); Govender, Brysiewicz and Bhengu (2015); Thopola, Kgole and Mamogobo (2013) and Ebrahimi et al. (2016) suggests that newly qualified professional nurses need support, approval and supervision until they gain independence. From the onset, it is expected that CSNs have the necessary skills that enable them to render professional and quality care within the clinical arena (Snell & Daniel 2014). In the literature, this is referred to as competence, and within the South African context, competence is defined as a cluster of related knowledge, skills, attitudes and values that enable an individual to effectively deliver career development services (Department of Higher Education & Training 2015). In this study, clinical competence refers to the CSN’s ability to integrate theory into practice by demonstrating knowledge, judgment and skill in order to render quality patient care (Hansen-Salie & Martin 2014).

### Problem statement

Clinical competence can be improved if effective supervision and mentoring are received during placement in healthcare facilities such as clinics and hospitals (Motala & Van Wyk 2016). There has been increased scrutiny into the roles of stakeholders, including professional nurses involved in the development of expert knowledge and skills of CSNs as these professional nurses are expected to provide such support throughout the 12 months of the period of community service. The question remains whether community service has an impact on CSN’s preparedness for the realities of dealing with the high burden of disease in the country.

Several studies including those of Tsotetsi (2012), Nkoane (2015), Zaayman (2016) and Govender, Brysiewicz and Bhengu (2017) have been conducted as the implementation of community service for nurses, but their focus was on the lived experiences of CSNs and the support they require during placement. Snell and Daniels (2014) conducted a study on professional nurses’ perceptions regarding CSN’s clinical competence that compared the clinical competence between degree and diploma graduate nurses in the Western Cape.

However, very little is known on how professional nurses perceive CSNs’ clinical competence in the North West province (NWP). The researcher identified a need to conduct a qualitative study exploring and describing the perceptions of professional nurses regarding the clinical competence of CSNs during their placement. This study provides some insight into these perceptions and identifies perceived problems that require improvement.

### Purpose of the study

The purpose of this study is to explore and describe the perceptions of registered professional nurses regarding the clinical competence of CSNs that they supervised in selected hospitals of the North West province, South Africa.

### Research question

What are the perceptions of professional nurses regarding CSNs’ clinical competence?

## Methodology

### Research setting

The study was conducted at two regional hospitals and one district hospital in the North West province, South Africa, where CSNs were allocated to do their 12 months of community service. Professional nurses were permanently employed at these hospitals and were supervisors for CSNs.

### Research design

This study followed a qualitative, exploratory, descriptive and contextual research design. According to Creswell and Creswell (2018), the qualitative research is a way of exploring and understanding the meanings that individuals or groups ascribe to a social or human problem. It is further stated that a qualitative design focuses on participants’ perspectives, their meanings and their multiple subjective views within their context and presents a complex, holistic picture (Creswell & Creswell 2018). In this study, the researcher aimed to acquire an understanding of professional nurses’ perceptions regarding the clinical competence of CSNs during their placement.

### Population and sampling method

The target population in this study was professional nurses in three selected hospitals in the NWP. The researcher obtained a list of units where CSNs were allocated during data collection from the hospital’s nursing services managers. The inclusion criteria were professional nurses with two or more years of experience after registering with SANC and who were directly responsible for supervising CSNs at the hospitals in NWP. Only the professional nurses who signed the informed consent participated in the study. The exclusion criteria were all operational managers because they have only indirect supervision of CSNs.

According to Gray and Grove (2020), the population is defined as all the components, including characters, objects or elements that meet certain criteria for inclusion in a particular world. The purposive sampling method was used to select participants in this study. The selection of individual participants was based on what was considered to be the representative of the population (Creswell 2014). According to Creswell and Creswell (2018), with purposive sampling, researchers select individuals who help them understand the research problem and the research question. Therefore, the researcher deliberately recruited participants who could best provide perceptions required on the competence of CSNs.

### Instrumentation for data collection

Face to face, focus group discussions (FGDs) with semi-structured, open questions were used to elicit the participants’ perceptions regarding the clinical competence of CSNs and to increase the credibility of the results (Creswell & Creswell 2018). Digital recording tape and field notes were used to record and capture verbal and non-verbal behaviours during the FGDs. The participants were asked the following two central questions:

- ‘What are your perceptions regarding the clinical competence of community service nurses?’
- ‘What suggestions do you have for improving the clinical competence of community service nurses during their placement?’

### Data collection process

The first author collected data through face-to-face FGDs between September and November 2018. It was easy to conduct face-to-face FGDs because that was before the coronavirus disease 2019 (COVID-19) pandemic and government regulations that emphasise social distancing. The first author is an experienced qualitative researcher with two publications and four citations. Three FGDs comprising groups of five participants each were held in the study. The discussions lasted between 30 and 60 min. Focus group discussions are defined as carefully planned discussions that take advantage of group dynamics to gain access to rich information in an efficient way (Grove, Gray & Burns 2015). According to Palmer (2019), FGDs are used for generating data on collective views and are useful in generating a rich understanding of participants’ perceptions.

The researcher had no personal relationship with the participants that might have inappropriately influenced them to participate in this study. However, being a former lecturer at the nursing education institution in the province, the researcher’s past personal and theoretical knowledge were bracketed so that full attention could be given to the perceptions of the professional nurses (Polit & Beck 2017).

The researcher recruited all participants after receiving written approval from the North-West University (NWU), North West Department of Health (DOH) and Chief Executive Officers (CEOs) of the hospitals where data were collected. The participants were recruited through consultation with the hospitals’ nursing service managers. After receiving the permission in writing to conduct the research from the selected hospitals, the researcher made an appointment to collect data through the nursing service managers, and data were collected on the days suggested and based on the availability of the participants. Before data collection, informed consent was obtained in writing by the researcher. The nursing service managers arranged quiet rooms, and the participants attended based on their availability and willingness to participate after the purpose of the research was thoroughly explained by the researcher. The participants had the right to refuse to participate or to withdraw from participating in the study without any punishment. The researcher conducted all face-to-face FGDs until data saturation was reached, meaning no new information was forthcoming (Creswell & Creswell 2018). This occurred after the third FGDs where no fresh insights emerged (Creswell & Creswell 2018). The researcher used a digital recorder to capture spoken words and field notes were taken during the FGDs. Later, the recording was transcribed verbatim by an independent person and verified by the researcher. Verified transcripts were then sent to the experienced researchers been the second and third authors for approval and prepared for analysis. An independent person took two full weeks to transcribe all FGDs. Both the first and the third authors included the field notes in the data analysis process.

### Data analysis

Data from the transcripts were analysed independently and manually by the first and the third authors, by following Pienaar’s four steps of data analysis (Pienaar 2017). The steps are as follows:

- **Step 1:** Data collection and analysis occurred simultaneously. ‘Basic concepts from the spoken words’ were identified (Pienaar 2017). Concepts were derived from the spoken words.
- **Step 2:** Similar concepts were grouped together. Constantly, as the concepts came up, the researcher separated and linked related concepts together.
- **Step 3:** The researcher identified new concepts, themes or clusters (insights). This level occurred as new information emerged during data collection, through intuitive deduction of the researcher.
- **Step 4:** The researcher built the storyline pattern to form a process and a generic framework for a unique African context. A consensus discussion was held by the first and the third authors to verify the final themes.

### Measures of trustworthiness

Trustworthiness was ensured by laying aside the researcher’s preconceived ideas about the professional nurses’ perceptions investigated and by following the criteria suggested by Lincoln and Guba (1985) cited in Mills and Birks (2014). Credibility was ensured as FGDs were carried out by the researcher herself and she is experienced in conducting these. Credibility was also ensured by checking the reliability of analyses with a qualified researcher who analysed the data independently and verified the research results. Dependability was ensured by supplying a detailed description of the research method and the appropriateness of the methodological applications. An audio recording was used, and an independent person transcribed raw data. The researcher validated the transcripts. Data were analysed independently by both the first and third authors. Existing literature was used for literature control. Transferability was ensured by purposively selecting participants based on their knowledge of the perceptions regarding the clinical competence of CSNs. Confirmability was achieved by ensuring credibility and transferability. Recording tape was used to record all participants’ responses.

### Ethical considerations

Ethical clearance for this study was granted by the North-West University Faculty of Agriculture, Science and Technology, Health Science Ethics Committee (reference number: NWU-00230-18-A9) and the three selected hospitals from North West province in South Africa. Professional nurses voluntarily participated in this study, and they were informed that they could withdraw their participation at any time, without penalty. Informed consent was obtained from the professional nurses after a thorough explanation of the study was given. The identity of all the professional nurses was protected by using card numbers as identity codes in lieu of names. All participants shared their perceptions without any judgment or coercion, and this ensured fairness during the participation.

## Results

Fifteen professional nurses participated in the study. This included 2 men and 13 women who supervised CSNs working in different wards of the selected hospitals in NWP. Twelve professional nurses completed their 4-year training following a diploma programme from nursing colleges and three followed a degree programme from a recognised university. Seven of the participants underwent community service themselves in different provinces of SA. The participants represented a single racial group (black) with age ranges of 28–61 years and having between 2 and 38 years of work experience after registration with SANC as professional nurses.

Three main themes and sub-themes emerged from the data analyses (see Table 1).

**TABLE 1:** Themes and sub-themes.

Themes	Sub-themes
1. Perceptions on clinical competence of CSNs	1.1. Perceived some CSNs to be competent and require minimum supervision
	1.2. Trusted with responsibilities
2. Challenges impacting on clinical competence of CSNs	2.1. Fear of taking responsibilities
	2.2. Failure to communicate
3. Suggestions to improve clinical competence of CSNs	3.1. Continuous support in specialised units and in-service training
	3.2. Encourage peer-group teaching
	3.3. Change of attitudes towards CSNs

CSNs, community service nurses.

### Theme 1: Perceptions on clinical competence of community service nurses

Perceptions on clinical competence of CSNs were the first theme identified in this study. Sub-themes for this are discussed below.

#### Sub-theme 1.1. Perceived some community service nurses to be competent and require minimum supervision

The majority of participants perceived some CSNs as competent in knowledge and skills. One of the participants said:

‘There are those that are very much competent that you can leave, and you know this person is having your back. When you are busy with something, this person is doing something, and she doesn’t wait for you to say do this.’ (Focus group discussion [FGD] 3, Participant [P] 5, female)

A majority of participants mentioned that most of the CSNs required minimum supervision but some thought that they would benefit from experienced guidance:

‘…they need lesser guidance. Most of them they have the confidence, because of usual they are based where there are no supervision, from my experience.’ (FGD2, P4, female)

‘They need those who are having experience just to guide. It doesn’t mean they don’t know.’ (FGD1, P5, female)

#### Sub-theme 1.2. Trusted with responsibilities

The participants of the study mentioned that some CSNs, whom they refer to as ‘commserves’, displayed high levels of commitment and could be trusted with responsibilities given to them. This is what participants said:

‘There are instances where the commserves were left to lead to the shift. That’s what I’m saying, in high care, we are working two/two. So, you will be with a commserve and you’ll be four professional nurse per shift with two staff nurses. So, somehow, during the course of time, we let them lead the shift.’ (FGD2, P1, female)

### Theme 2: Challenges impacting on clinical competence of community service nurses

Challenges impacting on clinical competence of CSNs were a second theme that emerged from the results of this current study. Sub-themes for this theme are discussed below.

#### Sub-theme 2.1. Fear of taking responsibilities

Some participants stated that a number of CSNs displayed fear and a lack of confidence when expected to do tasks that include allocation or delegation of duties to those nurses who have been working in the units for a long time. This is what a participant said about it:

‘The challenge that they face sometimes is that those people have been there for quite a long time. They (commserves) fear to delegate and do almost everything on their own without asking for help.’ (FDG 3, P2, female)

#### Sub-theme 2.2. Failure to communicate

Some participants mentioned that poor communication skills from the CSNs were the main challenge observed during the supervision of CSNs. This was evidenced by the following quotes:

‘So, somewhere, somehow, you’ll be just pushing, pushing, pushing and then there the eagerness of wanting to know or eng [*what*] won’t get too much of questions or too much of saying I want this… to know this and that. Wena [*you*] …you will be just following up and asking have you seen this? …just like that.’ (FGD2, P1, female)

### Theme 3: Suggestions to improve clinical competence of community service nurses

Suggestions to improve the clinical competence of CSNs were the third and last themes identified in the study. Sub-themes for this theme are supported with existing literature in the subsequent sections.

#### Sub-theme 3.1. Continuous support in specialised units and in-service trainings

A majority of participants suggested that there is a dire need for continuous support, especially in specialised medical units and provision of in-service training to improve the clinical competence of CSNs in such specialised units. Some participants suggested the following:

‘As much as we have specialised units and general units, in specialist units there’s always a challenge, and I think the only way of correcting it, like they would be allocated maybe in a specialised unit, we can to try and guide them, enough time to try and improve their skill where they lack.’ (FDG2, P2, female)

#### Sub-theme 3.2. Encourage peer-group teachings

The majority of participants suggested that peer teachings be encouraged amongst CSNs to improve their clinical competence. As one participant said:

‘The ones that are more competent, they can teach them. Even them, as community service nurses, they can communicate and give others more information, if they know more about it.’ (FDG3, P5, female)

### Sub-theme 3.3. Change of attitudes towards community service nurses

Some participants suggested that a significant number of supervisors need to improve their attitudes and actions towards CSNs in order to assist them to grow professionally. One participant said the following:

‘So, to ensure that these commserves, neh, they are competent enough, I think rona [*us*] as seniors, we should work on our attitude. Our attitude can help them to improve, to grow to be like…to perform well in communication and professional behaviour.’ (FDG2, P1, female)

## Discussion

### Perceptions on clinical competence of community service nurses

The main aim of this study was to explore and describe the perceptions of professional nurses regarding the clinical competence of CSNs under their supervision. The results of this study revealed that the participants had positive emotions with regard to how they perceive the clinical competence of CSNs. They perceived them (CSNs) as competent and requiring only a minimal supervision. These results are in contrast to those of a study conducted by Shezi (2014), in KwaZulu-Natal that revealed that not all CSNs were fully competent and independent to practise autonomously during their community service although some acquired some knowledge and skills. This concurs with the results of Makua (2016) that revealed that nurse managers perceived CSNs as different in their levels of competence. The study further reported that nurse managers perceived CSNs from the nursing colleges to be more competent than those from the university because of the unequal time allocation for clinical practice during their training. Furthermore, some participants indicated lack of trust in the education and training of newly qualified professional nurses who were engaged in community service. Qualification and the scope that the newly qualified professional nurses should cover were perceived as dubious by some participants in Makua’s study. This discrepancy also indicates that there is a need for more research into the quality of Nursing Education Institutions in the country. With regard to the amount of supervision required by CSNs, these perceptions contradict several studies conducted in South Africa including that of Nkoane (2015), Thopola et al. (2013), Khunou (2016), Makua (2016) and Tsotetsi (2012). The majority of participants in these studies reported that CSNs need efficient and dedicated supervision as they displayed minimum confidence and knowledge in the tasks assigned. This difference in perceptions indicates that more studies should be carried out to determine whether lesser or more supervision has any effect on the clinical competence of CSNs. These studies can be carried out with larger sample sizes for generalisation to the whole country.

### Challenges impacting on clinical competence of community service nurses

Fear of taking responsibilities particularly in undertaking new roles such as delegation of duties was reported as a challenge. Furthermore, the participants mentioned that CSNs were still behaving like students and could not do most of the duties without assistance of the supervisors. Missen et al. (2016) suggested that the ability to function independently and to be accountable for one’s own nursing practice are some of the competencies expected from the newly qualified nurse, which was not the case in this study. The results of this study concur with those of Magnusson et al. (2017) from England, which reports that many newly graduated nurses actively avoid delegating duties because of a lack of confidence. They often feel that healthcare assistants have more experience than them, and therefore they do not want to tell them what to do. However, Shipman, Hooten and Lea (2016) from Virginia found that participants felt confident transitioning to the role of registered nurses, making decisions and interacting with inter-professional staff after the completion of their residency programme. The difference in these results can logically be attributed to the newly qualified professional nurses’ varying and individual levels of confidence. This suggests a need to institute strategies that would support and build confidence in CSNs, and this could include a more robust and effective mentorship.

Failure to communicate was also cited as one of the challenges. The ability to communicate effectively is one of the valuable competencies needed for a newly qualified nurse (Missen et al. 2016). The perceptions of inability to communicate effectively amongst newly graduated nurses have been reported in previous studies Rhodes et al. 2013; Walker et al. 2013; Phillips et al. 2015) cited in Missen et al. (2016). The results of this current study confirm those from a qualitative study conducted in Iran by Ebrahimi et al. (2016), which reports failure to communicate amongst the obstacles raised by licenced nurses. This was established as a barrier in supporting newly graduated nurses in a clinical setting. According to Flott and Linden (2016), communication and interaction amongst staff members are important in the clinical environment. Therefore, failure to communicate not only impacts supervision but also compromises quality patient care. This suggests that CSNs might benefit from workshops aimed at improving their communication skills in the workplace.

### Suggestions raised to improve clinical competence of community service nurses

The participants suggested that CSNs need adequate support in terms of in-service training, peer-group teaching as well as change of supervisor’s attitudes. According to Pasila, Elo and Kääriäinen (2017), newly graduated nurses need more support to develop their competence. In the integrated systematic review conducted by Edward et al. (2017), authors suggested that support and positive relationships as well as working positively with experienced peers including mentors and nurses can have a meaningful influence on newly graduated nurses’ transition into practice (Edward et al. 2017). This will improve the confidence and competence of the newly graduated nurses. The same sentiments were shared by Zhang et al. (2016). According to Zhang et al. (2016), support strategies including mentoring contribute to the newly graduated nurses’ confidence and competence. The authors further mentioned that supporting newly graduated nurses had positive outcomes for all the stakeholders including the mentor, mentee and the organisation (Zhang et al. 2016). In the study conducted by Gardiner and Sheen (2016), the authors concluded that graduate nurses’ transitions are more likely to be positive when they are welcomed and supported by the professional nursing team. This is supported by Parker et al. (2014) who also assert that new graduate nurses experience positive transitions to nursing when they receive adequate and appropriate support in their new role and environment. All the above tally with the conclusions reached by Makua (2016) that newly qualified professional nurses are taught on the spot whilst working with experienced nurses as the learning opportunities arise, as well as by attending in-service programmes for all nurses. The participants in the current study further suggested that there is a need to encourage peer-teachings in the units to improve the clinical competence for CSNs. These results concur with those of a study conducted in Australia by Stone, Cooper and Cant (2013). The study revealed that peer learning improved participants’ self-confidence and was shown as acceptable to most of them. The results also concur with Stenberg and Calson (2015) who conducted a study in Sweden and substantially concluded that participants felt that when the preceptor was not physically present, it was perceived as a safe haven to have a fellow student with whom to discuss problems. The study further revealed that student nurses experienced a sense of increased learning as they felt more responsible when they took turns teaching each other in each instance ensuring they had the correct knowledge and skills, all of which were sufficient and up to date. According to Pålsson et al. (2017), participants who are given the opportunity to learn together with and from a peer during clinical practice education have improved levels of perceived self-efficacy. Furthermore, participants perceive their ability to perform nursing tasks in relation to their capacity in identifying and analysing patients’ care needs.

Change of attitude towards CSNs was also suggested by the participants. Thopola et al. (2013) concur that there is a need for stakeholders to professionally support CSNs. These researchers assert that in order for the relevant stakeholders to enable CSNs to deliver quality nursing and midwifery care, it is important to provide a positive and resourceful environment. They also add that improved attitudes of professional nurses would contribute positively to the competence of CSNs. Study by Freeling and Parker (2014), which focussed on ‘exploring experienced registered nurses’ attitudes, views and expectations of graduate nurses which may create a barrier for optimal graduate nurse performance’, concludes that nurse managers should be aware of the possible occurrence of unprofessional behaviour and increased workplace training and raise awareness regarding negative and unacceptable behaviour. It is therefore important for professional nurses to adjust their attitudes and treat CSNs as their colleagues and encourage a co-operative relationship and teamwork, which promotes commitment to the nursing profession. This is perceived as likely to enable CSNs, particularly those who lack confidence and feel more comfortable, which could eventually lead to them becoming more competent and confident practitioners.

## Limitations

This study used qualitative approach and focussed only on three hospitals in the NWP, SA that may decrease the chances of the generalisability of the results. However, parallels were found for many of the results of this study in research conducted in other SA provinces and also around the world. This suggests that many of the results cannot be considered unique to the NWP. Therefore, results obtained from this study could potentially be applied to other provinces of the country, hence a need for more research. to confirm these initial results.

## Recommendations

### For perceptions on clinical competence

It is recommended that workshops be arranged for professional nurses and topics such as staff management, ethical and professional behaviour be presented in an attempt to disseminate ideal standards. It is also recommended that professional nurses need to be reminded of the importance of their role as mentors in the midst of massive challenges. The CSNs are more likely to become good mentors in time if they themselves were mentored well. Further research studies are recommended to address the needs of professional nurses that would assist them in their mentoring role. It is also recommended that allocation for the mentoring role be done in a manner that CSNs are paired with experienced nurses who are likely to provide continuous guidance and support for the CSNs in becoming competent practitioners. These recommendations support the recommendation made by Govender et al. (2015).

### On challenges impacting on clinical competence

It was noted that some CSNs lack confidence and display a fear of taking responsibility that includes delegating tasks to other categories like Enrolled Nurses (ENs) and Enrolled Nurse Auxiliary (ENAs) with more years of experience. It is recommended that those CSNs be identified and allocated to someone experienced who would guide and assist them in dealing with these categories of nurses in terms of duty rosters and task delegations. It is also recommended that the hospital management look into staff recruitment and retention as this will not only improve the clinical competence of CSNs but also ensure improved patient care and thus relieve pressure on the current staff. It is further recommended that stakeholders develop sufficient scope of practice and job description for CSNs. This avoids confusion in the workplace and eliminates inconsistent working experiences for CSNs in different hospitals and possibly even in different wards. The study conducted by Govender et al. (2015) recommended that there should be a different job description for CSNs, which differs from that of the registered nurses.

### For improving clinical competence

Hospital management could improve clinical competence of CSNs by developing effective support systems that include proper orientation and mentoring programmes. This recommendation is in consistence with that of Khunou, (2016). The hospital training coordinator should assess the learning needs of the CSNs on a regular basis and arrange in-service training and continuous development programmes for them. A record of attendance must be kept as evidence. Possible strategies to improve professional nurses’ attitudes, communication skills and teaching roles could be researched and workshopped. The CSNs too could also benefit from guidance to improve their attitude, communication skills and ethical behaviour that would have a positive impact on their supervision. It is also recommended that both professional nurses and CSNs establish teamwork and collaboration set to improve the supervision process boost confidence and subsequently improve CSNs’ clinical competence.

## Conclusion

The focus of this study was on exploring and describing the perceptions of professional nurses regarding the clinical competence of CSNs in the NWP, SA. It is suggested that even though the majority of CSNs were perceived to be competent and capable of working independently, they still required supervision and mentorship to refine their competency. Furthermore, the study reported similar challenges noted from previous studies that were perceived to be affecting CSNs’ ability to deliver quality health care and recommendations for improvements were made. Paying attention to measures for improving clinical competence of CSNs as well as dealing with the challenges affecting their clinical competence will be of great benefit to the healthcare facilities to ensure quality nursing care. The results of this qualitative study add important information to the body of knowledge in nursing education and clinical practice, which might not have been achieved if other approaches had been used.
